# Innate immune recognition and evasion strategies of hepatitis B virus: from DNA to RNA and viral proteins

**DOI:** 10.3389/fimmu.2026.1741620

**Published:** 2026-03-03

**Authors:** Zhenghao Chen, Rui Hu, Huajun Ye, Qiuxun Chen, Yuhang Liu, Xinyu Zhang, Yingting Chen, You Wu, Ciliang Jin

**Affiliations:** 1 Alberta Institute, Wenzhou Medical University, Wenzhou, China; 2 Department of Gastroenterology and Hepatology, The First Affiliated Hospital of Wenzhou Medical University, Wenzhou, China

**Keywords:** cccDNA, cGAS–STING, HBx, hepatitis B virus, immune evasion, innate immunity, PRRs, RIG-I/MAVS

## Abstract

Innate immunity constrains the hepatitis B virus (HBV) by sensing pathogen-associated molecular patterns (PAMPs) and inducing type I/III interferons and interferon-stimulated genes. This review synthesizes molecular mechanisms by which HBV nucleic acids and proteins are detected by pattern recognition receptors (PRRs) and how the virus evades such surveillance. At the DNA level, covalently closed circular DNA (cccDNA) persists as a chromatin-like episome with low immunogenicity; cGAS–STING signaling is functionally dampened, whereas nuclear interferon-inducible protein 16(IFI16) and cytoplasmic/nuclear ABCF1 bind cccDNA to repress transcription, and APOBEC3A-mediated deamination requires robust interferon signaling. At the RNA level, TLR3/7/8 and retinoic acid-inducible Gene I(RIG-I) sense circulating HBV RNA and 5′-triphosphate pregenomic RNA, respectively. HBV counteracts RIG-I-like receptor (RLR) pathways through ADAR1 editing, TIAR-dependent translational control, and a metabolic checkpoint involving lactate-MAVS/hexokinase, whereas spliced viral RNAs (svRNAs) have emerged as immunologically relevant species. At the protein level, Hepatitis B Surface Antigen (HBsAg) impairs interferons (IFN) induction by blocking the TAK1–TAB2–NF-κB/IRF axis; Hepatitis B Virus X Protein (HBx) sustains cccDNA transcription via DDB1-directed Smc5/6 degradation and broadly suppresses PRR/IFN signaling, with TRIM25 acting as a host restriction factor. These insights nominate combinatorial strategies—PRR agonists (TLR/STING), MAVS sensitization, metabolic disinhibition, pharmacological disruption of the HBx–DDB1 axis, and reinforcement of IFI16/ABCF1—to achieve functional control of cccDNA and advance curative hepatitis B virus (HBV) therapy.

## 1 Introduction

Hepatitis B virus (HBV) infection remains a global health issue, affecting an estimated 254 million people globally and resulting in nearly 1.1 million deaths each year. The Western Pacific and African regions bear the highest disease burden, highlighting ongoing disparities and the urgent need for enhanced prevention and treatment strategies aligned with WHO sustainability goals (1, 2). It is a member of the Hepadnaviridae family and primarily infects hepatocytes, leading to both acute and chronic liver inflammation. The virus is transmitted through blood, sexual contact, and from mother to child during birth. Understanding the molecular mechanisms behind HBV infection, immune evasion, and pathogenesis is critical for developing effective therapeutic strategies (3).

Hepatitis B virus (HBV) replication follows a complex, multi-step cycle that is central to its persistence and chronicity. Upon entry into hepatocytes through the sodium taurocholate co-transporting polypeptide (NTCP) receptor, the virus releases its relaxed circular DNA (RC DNA), which is subsequently converted into covalently closed circular DNA (cccDNA). This cccDNA functions as a stable, episomal template for the transcription of various viral RNAs, including the pregenomic RNA (pgRNA). The pgRNA is then encapsulated into core particles, where it undergoes reverse transcription to generate progeny rcDNA. These newly formed core particles can either be enveloped for virion secretion or recycled back to the nucleus to amplify the cccDNA pool, thereby facilitating the perpetuation of chronic infection (4). This replication cycle underscores the pivotal role of cccDNA in HBV’s immune evasion strategies, directly linking to the innate immune pathways discussed in subsequent sections.

The innate immune response serves as the host’s first line of defense against viral infections, and its activation depends on the recognition of pathogen-associated molecular patterns (PAMPs) (5, 6). Specialized pattern recognition receptors (PRRs) identify virus-specific molecular structures and trigger cascade reactions, inducing the production of type I and III interferons (IFN-I/III) (7–10). Through autocrine and paracrine mechanisms, interferons stimulate the expression of interferon-stimulated genes (ISGs), which restrict viral replication within host cells and play crucial roles in initiating innate immune responses (11). Therefore, a deeper understanding of the molecular basis by which the host innate immune system recognizes and responds to viral infections is essential for elucidating viral transmission and pathogenesis and developing effective therapeutic interventions (12–14). Studies have shown that PRRs responsible for recognizing viral nucleic acids mainly include Toll-like receptors (TLRs), RIG-I-like receptors (RLRs), and cytosolic DNA sensors (15–17). Activation of these PRRs further induces robust IFN-I/III antiviral responses, promoting the expression of multiple ISGs (e.g., APOBEC3G, IFITM family, and MX1) to suppress viral replication and spread (11, 18).

However, hepatitis viruses have evolved various mechanisms to evade or interfere with the host innate immune surveillance. For instance, HBV covalently closed circular DNA (cccDNA) effectively circumvent innate immune recognition and inhibit interferon signaling pathways (12, 13). Thus, investigating the molecular mechanisms of PRR-mediated viral recognition, signal transduction pathways, and viral immune evasion strategies during hepatitis virus infections is critical for understanding the mechanisms of viral chronicity and guiding the development of novel therapeutic approaches (e.g., TLR agonists or IFN combination therapies) (19, 20).

This review summarizes recent advances in our understanding of how host PRRs recognize HBV and initiate innate immune responses. It delves into the molecular mechanisms and regulatory networks underlying key receptor activation and outlines viral strategies for innate immune evasion. We anticipate that these insights will provide new research perspectives and therapeutic targets for the prevention and treatment of viral hepatitis.

**Figure 1:**
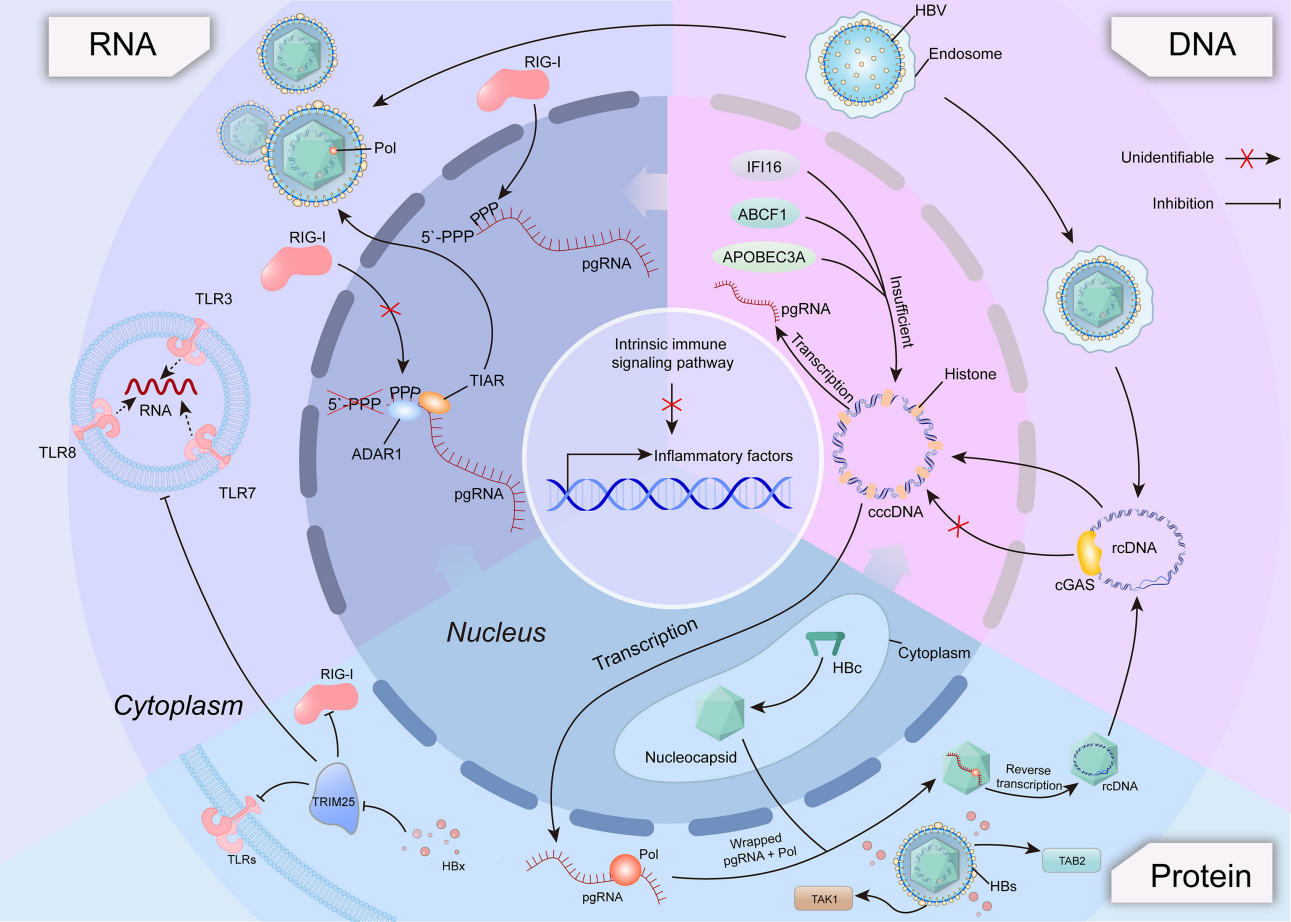
This figure depicts how hepatitis B virus (HBV) engages with host innate immune pathways through its DNA, RNA, and viral proteins, highlighting multiple immune evasion strategies. On the DNA axis, cytosolic DNA sensors such as cGAS recognize rcDNA but fail to detect cccDNA due to its chromatinized nuclear localization, whereas IFI16, ABCF1, and APOBEC3A exhibit partial recognition of cccDNA. On the RNA axis, HBV-derived RNAs are sensed by TLR3/7/8 and RIG-I, with viral RNA modifications mediated by host ADAR1 and TIAR aiding immune escape. On the protein axis, HBc assembles into nucleocapsids enabling rcDNA formation via reverse transcription, while viral proteins HBs and HBx interfere with innate signaling by targeting TAK1/TAB2 and TRIM25, respectively. Together, these interactions illustrate the multi-layered mechanisms by which HBV evades innate immune surveillance.

In order to provide a clear overview of the study’s findings, one table (**Table 1**) and one figure (**Figure 1**) present a comprehensive summary of the key mechanisms and interactions involved in the immune response to hepatitis B virus, which are discussed throughout the manuscript.

**Table 1:** Innate sensors of hepatitis viruses and downstream signaling.

Sensors	PAMP	Signal axis	Cell type used	Infection outcome	Immune effector response	Reference
cGAS	rcDNA, cccDNA	→ Cgamp → STING → TBK1 → IRF3 → NF-kB	Human Hepatoma Cell Line (HepG2), Primary Human Hepatocytes, Murine Hepatoma Cell Line (AML12)	Inhibits cccDNA-driven transcription	Type I/III IFNs; ISGs; NF-xB-driven cytokines	(21–26)
IFI16	cccDNA	→ STING → TBK1 → IRF3	Human Hepatoma Cell Line (HepG2, Huh7), Primary Human Hepatocytes	Inhibits cccDNA-driven transcription	Type I IFNs; ISGs; HBV cccDNA/HBsAg/HBeAg repression	(27, 28)
ABCF1	cccDNA	→ STING → TBK1 → IRF3	Human Hepatoma Cell Line (HepG2, HLCZ01, Huh7)	Inhibits cccDNA-driven transcription	Type I/III IFNs; ISGs; HBV cccDNA/HBsAg/HBeAg/HBc repression	(29)
APOBEC3A	cccDNA	Induced by the IFN-JAK/STAT axis; Act directly as an effector enzyme	Human Hepatoma Cell Line (HepaRG, HepG2), Primary Human Hepatocytes	Decrease cccDNA levels	Cytidine deamination of cccDNA → degradation; antiviral restriction	(30, 31)
TLR3	dsRNA	→ TRIF → TBK1/IKKϵ → IRF3/7 & NF-kB	Human Hepatoma Cell Line (dHepaRG), Primary Human Hepatocytes, Primary Murine Hepatocytes	Inhibits HBV RNA transcription, Promotes HBV RNA degradation	Type I IFNs; ISGs; TNF, IL-1/6, CXCL10; cccDNA/HBsAg/HBeAg repression	(32–34)
TLR7	ssRNA	→ MyD88 → IRAK4/1 → TRAF6 → NF-kB & IRF7	Human Hepatoma Cell Line (HepG2), AAV/HBV Murine Model	Inhibits HBV Replication, Decrease Viral proteins levels	Strong Type I IFNs/ISGs; TNF, IL-6; Activate adaptive immunity	(35–42)
TLR8	ssRNA	→ MyD88 → IRAK4/1 → TRAF6 → NF-kB & IRF7	Primary Human Hepatocytes, HBV Woodchuck Model, AAV/HBV Murine Model, HBV Cynomolgus macaques Model	Inhibits HBV Replication, Decrease Viral proteins levels	moderate IFN/ISGs; TNF, IL-12, IL-6; Activate adaptive immunity	(40, 43–47)
RIG-I	pgRNA	→ MAVS → TBK1/IKKϵ → IRF3/7 & NF-kB	Human Hepatoma Cell Line (HepaRG, HepG2), Primary Human Hepatocytes	Decrease cccDNA levels	Type I/III IFNs; ISGs	(48–59)

## 2 Sensing of HBV DNA

Covalently closed circular (cccDNA) is a stable mini-chromosome formed by HBV in the nuclei of infected liver cells, serving as a template for viral RNA transcription and supporting the synthesis of viral proteins and genome replication (60–62). The long-term retention of cccDNA is the fundamental cause of HBV chronicity. It can stably exist in the host cell nucleus for several months (with a half-life of approximately 40 days), maintaining an infected state through intracellular genomic recovery, and a continuous replenishment pool (pool) of secondary infection (63–65). Because cccDNA is located in the cell nucleus and has a stable chromatin structure, existing drugs find it difficult to directly target and eliminate it, which has become the main obstacle to the radical cure of HBV (66, 67). cccDNA is formed by relaxed circular DNA (rcDNA) (68), rcDNA contains an incomplete double-stranded structure, and existing evidence indicates that its broken ends need to be repaired by the host DNA repair system (such as ligase, polymerase, etc.) to form a covalently closed circular structure (60, 69–71). The complete set of host factors has not yet been fully elucidated yet (60). The level of serum HBV DNA not only includes cccDNA and rcDNA but also Double-stranded Linear DNA (dslDNA). It can originate from abnormally processed rcDNA or by-products during the infection process, plays a secondary role in viral infection and pathological mechanisms (72).

cccDNA is located within the nucleus of infected hepatocytes and forms a microsomal structure, which is assembled into chromatin by host histones and non-histones. This structure is similar to that of host DNA, making cccDNA less likely to be recognized by the immune system as a foreign substance (73–75). The nuclear localization of cccDNA makes it a difficult target for antiviral and immune responses (66, 76), thereby avoiding direct detection and clearance by the innate immune system. The actively transcribed cccDNA forms an interaction network with the host genome, further strengthening its “pseudo-host” attribute and preventing it from being cleared (77). Furthermore, the activity of the DNA innate immune sensing pathway in liver cells is low. This inherent poor reactivity makes hepatocytes lack a strong innate immune recognition ability even when facing exogenous DNA such as HBV cccDNA (21, 78).

Cyclic GMP-AMP synthase (cGAS) is a crucial cytosolic DNA sensor that plays a vital role in innate immunity and cellular processes. cGAS detects both foreign and self-DNA, producing cGAMP to activate the STING pathway and trigger immune responses (22–24). As a cytoplasmic DNA sensor, cGAS can recognize naked relaxed circular HBV DNA (rcDNA) *in vitro*, activating the cGAS-STING pathway to induce innate immune responses (25, 26, 79). When the cGAS pathway is forcibly activated, the intracellular level of cccDNA can be significantly reduced, indicating that cGAS has the potential to inhibit cccDNA (80). However, the immune escape mechanism of HBV hinders this process. Experiments have shown that in cell culture models and humanized mice, HBV infection inhibits the activity and expression level of cGAS, thereby weakening the host’s immune surveillance of the virus (27).

It has been discovered that interferon-inducible protein 16 (IFI16) is a unique intrinsic immune sensor in the nucleus that is capable of recognizing and binding to HBV cccDNA within the liver cell nucleus. Targeting the interferon-stimulated response element (ISRE) in cccDNA promotes epigenetic inhibition (such as promoting inhibitory histone modification). Therefore, inhibiting the transcription of cccDNA and replication of HBV (28, 29). Although IFI16 can recognize cccDNA and initiate inhibition, the persistent presence of cccDNA itself indicates that this recognition mechanism is insufficient to completely eliminate it in the *in vivo* environment, or that there are pathways to evade the continuous action of this mechanism. This may be related to the expression regulation of IFI16 itself, the protective structure of cccDNA microchromosomes, or the interference of other host/viral factors (66).

A recent study by Ren et al. identified ATP-binding cassette subfamily F member 1 (ABCF1) as a novel host restriction factor that directly binds HBV covalently closed circular DNA (cccDNA). ABCF1 recognizes cccDNA via its KKx4 motif and undergoes PolyQ-mediated phase separation, which both activates type I/III interferon responses and inhibits HBV transcription by preventing RNA polymerase II recruitment. HBV counteracts this defense by HBx-mediated ubiquitination and degradation of SOX4, thereby suppressing ABCF1 transcription (30). These findings reveal a previously unrecognized innate immune mechanism against cccDNA and highlight ABCF1 as a potential therapeutic target for HBV infection. Moreover, Stadler et al. (2021) discovered that the deamination of cccDNA mediated by APOBEC3A, an important DNA cytosine deaminase in the human body, requires the induction of interferon signaling (31, 81). As demonstrated by Julie Lucifora et al. (2014), APOBEC3A, along with APOBEC3B, is essential for the interferon-α or LTβR agonist-induced degradation of HBV cccDNA (82). These molecules target cccDNA in the nucleus, catalyzing cytidine deamination and triggering irreversible degradation of cccDNA without harming the host genome. This process provides a promising basis for new therapies aimed at curing hepatitis B.

In conclusion, HBV cccDNA largely escapes detection by key DNA sensors, with pathways such as cGAS–STING being functionally suppressed during infection. Sensors, such as IFI16 and ABCF1, can recognize cccDNA, but their antiviral effects are insufficient for clearance, as HBV actively limits their activity. Clearance mechanisms requiring strong interferon signaling, such as APOBEC3A-mediated deamination, are further hindered by the weak initial recognition of cccDNA, enabling viral persistence.

## 3 Sensing of HBV RNA

### 3.1 Recognition of HBV RNA by toll-like receptors

cccDNA serves as the transcriptional template for six principal HBV RNAs—preC, pg, preS1, preS2, S, and HBx transcripts. These viral RNAs possess a common 3′ terminus and exhibit overlapping sequences to varying extents (83). Circulating HBV RNA (cirB-RNA) is a recently identified serum biomarker that reflects cccDNA transcriptional activity, with its levels showing a strong correlation with intrahepatic transcriptional activity (84).

Toll-like receptors (TLRs) are crucial components of the innate immune system, recognizing conserved molecular patterns from pathogens and initiating immune responses (85). TLRs are transmembrane proteins with leucine-rich-repeat motifs for ligand binding and a cytoplasmic domain for signaling (86). TLRs (Toll-like receptors) recruit adaptor proteins (such as MyD88 and TRIF) in specific cellular compartments, such as the plasma membrane or endosomes, assembling supermolecular ordered centers (SMOCs) and initiating signal cascades. These signaling pathways activate transcription factors such as NF-κB, AP-1, and interferon regulatory factors (IRF), inducing the expression of immune genes such as inflammatory cytokines and interferons, thereby driving inflammatory responses and connecting to adaptive immune responses (32, 33, 85).

Members of the TLR family play heterogeneous roles in HBV infection, and some subtypes included TLR3,7,8 are particularly important in virus detection or immune regulation.

TLR3 specifically recognizes double-stranded RNA (dsRNA) (34). In a murine *in vivo* model of chronic HBV replication, delivering the TLR-3 ligand poly(I:C) to the liver via F4/80-targeted calcium phosphate nanoparticles resulted in efficient uptake by relevant hepatic cells and a robust induction of innate antiviral cytokine responses, accompanied by marked reductions in HBsAg, e antigen(HBeAg), and HBV DNA (35).

Corroborating these findings, screening in human hepatocyte *in vitro* models identified Pam3CSK4 (a TLR1/2 ligand) and poly(I:C)-(HMW) (a TLR3/MDA5 ligand) as the most potent suppressors across multiple HBV readouts with minimal viral rebound upon treatment withdrawal; the more TLR-3–specific agonist Riboxxol likewise exhibited strong activity in PHH, suggesting a sustained impact on cccDNA (36, 37).

In recent years, TLR7 has become a major research focus. It is an endosomal PRR that senses single-stranded RNA (ssRNA) (38, 39). Its degradation products via a dual-pocket mechanism—one pocket binding guanosine/derivatives and the other recognizing uridine-rich ssRNA fragments—while endogenous RNA modifications (e.g., pseudouridine) attenuate activation (40, 41). After TLR7 is activated, it triggers the production of interferons (IFN) and inflammatory cytokines through the MyD88-dependent signaling pathway, thereby inhibiting HBV replication (42). The stimulation of TLR7 agonists can enhance the host’s innate immune response, leading to the release of antiviral cytokines and inhibiting HBV replication in experimental models (43–45). TLR8 is similar to TLR7 and detects the viral ssRNA components (46, 47). Studies have shown that TLR8 agonists can activate downstream signaling pathways in immune cell models and reduce HBV DNA levels, indicating their direct antiviral effect (43, 87, 88). However, HBV infection can lead to a decrease in TLR8 expression or disruption of its signaling. In patients treated with peg-IFN-α, the partial restoration of TLR8 function is associated with viral suppression, highlighting the potential of TLR8 as a key point in innate immunity regulation (89).

In addition to the above three types of TLRs, although TLR2 and TLR4 do not recognize RNA, the research related to their interaction with HBV infection still provides us with a new perspective. In primary human hepatocytes *in vitro*, TLR-2 recognizes HBV particles and activates pro-inflammatory signaling, thereby inducing an anti-HBV innate immune response (90, 91). The activation of TLR2 is not only a trigger for innate immunity, but also indirectly enhances the HBV-specific T cell response through cytokine-mediated signals, which is helpful for clearing the virus (92, 93). This demonstrates how innate immunity “connects” with adaptive immunity, strengthening the overall antiviral defense. TLR-4, critical in innate immunity (94, 95), in patients with chronic hepatitis B (CHB), its expression—including serum levels—is markedly increased and positively associated with HBV DNA burden and liver injury indices (ALT, AST), supporting its potential application as a diagnostic and predictive biomarker of disease activity (96, 97). By activating CD11b&^+^ myeloid cells, TLR4 signaling promotes viral clearance; in C57BL/6 mice it drives their proliferation and differentiation and the interferon-mediated, direct suppression of HBV replication, underscoring the central role of innate immunity (98). A latest study found that HBV exploits its antigens (e.g., HBsAg) to disrupt TLR4 signaling and evade innate immune surveillance, weakening innate immunity and fostering chronic persistence (48).

Although TLR3, TLR7, and TLR8 are canonical nucleic acid PRRs, there is currently no definitive evidence that they directly recognize HBV-derived RNA during natural infection; instead, HBV largely escapes surveillance via nucleocapsid shielding and viral antagonism, leaving viral RNA poorly sensed. Thus, current approaches mainly employ exogenous agonists to artificially activate TLR signaling and elicit IFN-α/NF-κB–driven antiviral programs that indirectly restrict HBV replication (35, 43, 45, 87, 88). Similarly, TLR2 signals through the TLR1/2–MyD88–NF-κB axis and requires Pam3CSK4 to initiate antiviral responses (36, 37). Overall, these pathways suppress cccDNA transcriptional output and accelerate the decay of intracellular HBV RNA, particularly pgRNA, thereby compromising viral RNA stability.

### 3.2 Recognition of pregenomic RNA by RIG-I and relevant evading strategies

Pregenomic RNA (pgRNA) is a pivotal molecule in the HBV replication cycle and also functions as a potent PAMP in innate immune recognition. Synthesized in the nucleus from covalently closed circular DNA (cccDNA), pgRNA is exported to the cytoplasm, where it serves two critical roles: (1) as the template for reverse transcription to generate new viral DNA genomes, and (2) as the mRNA for translation of the HBV core protein (HBcAg) and polymerase (Pol) (49, 50).

RIG-I is a crucial innate immune receptor that detects viral RNA and triggers antiviral responses (51). It distinguishes viral RNA from host RNA through specific conformational changes and recognition of unique features such as 5’-triphosphate dsRNA blunt ends (52, 53). RIG-I employs multiple proofreading mechanisms, including an intrinsic gating mechanism and ATPase activity, to ensure accurate viral RNA detection while avoiding responses to host RNAs (53). Upon binding viral RNA, RIG-I releases its signaling domains (CARDs), which interact with mitochondrial antiviral signaling protein(MAVS) to initiate interferon production (54, 55). Recent research shows that RIG-I signaling occurs rapidly and efficiently using the constitutively expressed receptor pool, without mass aggregation at the mitochondrial membrane (56). This rapid signaling relay allows for an efficient antiviral response, highlighting RIG-I’s critical role in host defense against RNA viruses.

Structurally, pgRNA retains a 5’-triphosphate (5’-ppp) end, a molecular signature absent in host mRNAs, making it a strong ligand for retinoic acid-inducible Gene I (RIG-I), a cytosolic RNA sensor. RIG-I recognizes this 5’-ppp moiety and initiates antiviral signaling via MAVS, leading to type I interferon and pro-inflammatory cytokine production (57–59). It not only suppresses HBV replication but also impairs *de novo* cccDNA formation while promoting the decay of established cccDNA (99).

Host cells prevent aberrant immune activation through specific RNA modifications: Endogenous mRNAs evade RIG-I detection by structurally modifying their 5’ termini with m7G caps and 2’-O-ribose methylation. These modifications mask the 5’-triphosphate double-stranded (5’ PPP dsRNA) signatures characteristic of viral RNAs, thereby blocking RIG-I’s recognition capacity and preventing detrimental autoimmune responses that would otherwise compromise cellular homeostasis (58). Similarly, HBV may use modifications such as ADAR1-mediated A-to-I editing and TIAR to mask 5’ppp or dsRNA features altering the structure of pgRNA and reduce RIG-I activation (100).

The role of ADAR1(protein encoded by human gene) in hepatitis B virus (HBV) infection is complex and controversial. Wang et al. (2021) and Li et al. (2021) demonstrated that ADAR1 helps HBV evade immune recognition by editing viral RNA, IFN-α induces ADAR1 to suppress MAVS expression through RNA editing and HuR-dependent mRNA destabilization, thereby dampening antiviral signaling. This suppression is counteracted by HBc-SP1 inhibition of MAVS transcription. CHB patients carrying the rs3746662A allele exhibit higher MAVS expression and enhanced IFN-α responsiveness, highlighting MAVS agonism as a potential strategy to improve IFN-α efficacy in HBV therapy (100, 101), while Yang et al. (2022) and Liu et al. (2019) indicated that metabolic stress may invert ADAR1 Function. In HBV Infection In CHB with NAFLD comorbidity, ADAR1 transitions from a viral facilitator to an HBV suppressor, as evidenced by >60% ADAR1 downregulation in patient livers. Palmitic acid-treated hepatocytes confirm ADAR1 gain/loss inversely regulates HBV DNA replication via miR-122 induction, countering lipotoxicity (102, 103).

TIAR, a novel cellular factor, widely expressed in mammalian cells, regulates HBV replication by binding to the 5’ ϵ element of pgRNA to control the translational equilibrium of the core protein (Cp) and DNA polymerase (Pol). Induced by Cp, TIAR translocates from the nucleus to the cytoplasm, where this relocation indirectly represses Pol translation. This TIAR-mediated pathway ensures the precise balance of Cp and Pol expression essential for efficient viral replication within infected hepatocytes (104). Although there is no clear evidence that TIAR can reduce the activation of RIG-I, current data support that its primary contribution to the HBV life cycle is to reshape the translational output of pgRNA. Given that both ADAR1 and TIAR are involved in the post-transcriptional regulation of HBV RNA, a functional synergy may exist between these two factors, offering a potential direction for future research into the coordinated control of HBV replication and host immune evasion. Future studies should elucidate the precise molecular mechanism by which cytoplasmic TIAR represses Pol translation and explore TIAR’s potential as a novel therapeutic target to disrupt HBV replication and restore host immune recognition.

In addition to changing pgRNA, HBV directly inhibits the RIG-I-mediated immune response through multiple molecular mechanisms to ensure persistent infection. HBV activates glycolysis to produce LDH-A-derived lactate, which directly binds MAVS and disrupts its mitochondrial aggregation, thereby suppressing RIG-I/RLR signaling and interferon production. Concurrently, HBV sequesters MAVS via hexokinase (HK)-mediated ternary complex formation, establishing a metabolic checkpoint for immune evasion (105).

### 3.3 HBV splice variants RNA: an emerging research focus

As we mentioned above, the core genome of HBV consists of a 3.5 kb pgRNA, which not only serves as a template for viral DNA replication but also generates multiple Splice Variants RNA (svRNA) through splicing mechanisms of host cells (100, 101). The splicing of HBV pgRNA can generate at least 20 different splice variants RNA (106), accounting for 13-28% of the total viral RNA (107). The most common types are single-splice SP1 and SP9 (107–109). Various splice variants could be detected in independent biological repeat experiments as well as in liver biopsy samples, serum samples of patients and primary human liver cells infected with HBV, demonstrating their biological relevance (107, 110).

Evidence indicates that non-canonical proteins encoded by svRNAs, notably HBSP and RT’-RH, act as potent IFN antagonists. By suppressing STAT1 phosphorylation and impeding STAT1/2 nuclear translocation, these proteins downregulate the JAK-STAT pathway. This dual blockade attenuates the transcription of antiviral effectors (e.g., OAS1), enabling viral escape and contributing to IFN resistance (110–112).

Furthermore, HBV splicing is strictly orchestrated by host machinery and viral cis-acting regulatory elements. HBV RNA splicing is closely regulated by the host cell splicing mechanism and is controlled by HBV’s own post-transcriptional regulatory RNA elements of (107). The splicing efficiency is cell type-dependent. It is relatively high in human liver cancer cells (such as Huh7 and HepG2), but lower in human hepatic stellate cells, mouse liver cancer cells, or human non-liver-derived cells (113). This suggests that splicing may be one of the mechanisms that limits the host range of HBV. Overall, RNA splicing is not necessary for HBV replication (114). Although the mechanisms and functions of HBV svRNAs remain incompletely elucidated, they have emerged as a prominent focus of HBV research, offering new perspectives for advancing our understanding of HBV biology and pathogenesis and for identifying potential biomarkers and therapeutic targets (114).

## 4 Sensing of HBV protein

The key proteins encoded by hepatitis B virus (HBV)—including the core protein (HBc/Cp), the surface antigen proteins (HBsAg), the transcriptional regulator X protein (HBx) and the e antigen (HBeAg). These proteins can modulate innate immune sensing receptors as well as interferon signaling pathways, thereby enabling HBV to evade immune clearance and establish persistent infection.

### 4.1 Hepatitis B core antigen and hepatitis B e antigen

HBc is a multifunctional protein composed of 183 amino acids, translated from pgRNA (115, 116). Latest studies have discovered that its structure contains two major functional domains: N-terminal domain (NTD) and C-terminal domain (CTD). NTD is responsible for the self-assembly of the capsid. The HBc protein forms homodimers through NTD and further assembles into an icosahedral symmetric viral capsid. These capsids are usually composed of 90 to 120 dimers, including two configurations: T = 3 (90 dimers) and T = 4 (120 dimers) (117, 118). CTD is rich in arginine residues and mainly functions in binding to genomic RNA (pgRNA), participating in the packaging and reverse transcription of viral nucleic acids, connected to the NTD through a flexible linker region (119, 120). Furthermore, CTD undergoes dynamic phosphorylation and dephosphorylation modifications: Phosphorylation (such as at the Ser162 and Ser170 sites) promotes the binding of RNA to DNA polymerase, facilitating the initiation of viral replication; as the capsid matures, dephosphorylation helps with the release of the virus (119, 121).

The direct recognition of HBcAg by the innate immune system remains unclear. However, the capsid structure formed by HBcAg in the cytoplasm is the main target of T-cell-mediated immune responses, and plays a crucial role especially in the process of eliminating infected liver cells (116, 122). As we mentioned, cccDNA persists in the nuclei of liver cells (28), HBcAg, as the core protein for nucleocapsid assembly, indirectly maintains the stability of cccDNA (123).

Hepatitis B e Antigen (HBeAg) has a high degree of homology with HBcAg, sharing the same core domain (124, 125). The research on the role of HBeAg in influencing the innate immunity is relatively unclear. Instead, its characteristics as a serum marker and an intrahepatic marker can be used as endpoints for treatment and to predict a long-term outcome (126–128).

### 4.2 Hepatitis B surface antigen

HBsAg proteins are the key proteins on the HBV viral envelope and a key molecule for HBV virus attachment to host cells and serve as the main target of Neutralizing Antibodies (NAbs) (123). They include three major subtypes: large hepatitis B surface protein (LHBs), middle hepatitis B surface protein (MHBs), and small hepatitis B surface protein (SHBs) (129–131). LHBs are translated from 2.4-kb RNAs (132), while MHBs and SHBs are translated from 2.1-kb RNAs (132, 133). The high-resolution structure of the HBsAg protein and its assembly mode on the viral envelope remain unclear, which limits a deeper understanding of its function (134). HBsAg significantly inhibits the production of type I interferon (IFN-I) by interfering with the innate immune signaling pathway. The specific mechanism involves HBsAg directly binding to TAK1 (TGF-β -activated kinase 1) and TAB2 (TAK1-binding protein 2), hindering the phosphorylation of TAK1 and the K63 junction polyubiquitination of TAB2, thereby blocking the activation of the NF-κB pathway (135). HBsAg also enhances the phosphorylation of TBK1 (TANK-binding kinase 1), but simultaneously inhibits the function of interferon regulatory factors, resulting in the obstruction of type I interferon production (136).

### 4.3 Hepatitis B X protein

Hepatitis B X protein (HBx) is translated from 0.7-kb RNAs (137, 138). In chronic hepatitis B virus (HBV) infection, the X protein (HBx) plays a central regulatory role in both viral replication and host cell transformation. HBx is crucial for the transcription of HBV cccDNA, and it maintains viral replication by promoting the transcription of the cccDNA mini-chromosomes (139). HBx drives HBV replication and oncogenesis by binding DDB1 to degrade the Smc5/6 restriction complex. Losing Smc5/6 both unleashes cccDNA transcription—sustaining viral RNA/antigen production—and impairs homologous recombination repair, causing DNA damage and promoting hepatocellular carcinoma (HCC) (140). A pharmacological study further indicates that nitazoxanide (NTZ) can block the HBx–DDB1 interaction and restore Smc5/6 levels, thereby suppressing HBV transcription and viral protein expression while alleviating HBx-induced DNA-repair defects and transformation risk (141).

HBx mainly affects the innate immunity through mechanisms: inhibition of the interferon signaling pathway and disruption of the pattern recognition receptor signaling pathway. HBx targets interferon-stimulated genes (ISGs), directly binding and inhibiting their antiviral functions (such as TRIM25), weakening the antiviral effect of IFN on HBV (142–144), down-regulating mitochondrial antiviral signaling protein (MAVS), blocking the induction of IFN-β, and disrupting the subsequent adaptive immune response (145). HBx also inhibits the innate immune response by suppressing key molecules of the PRR pathway such as TLR/RLR/NLR(such as RIG-I), blocking the activation of downstream interferon regulatory factors (IRF) and NF-κB (146). TRIM25 is a protein with E3 ubiquitin ligase activity (147, 148), degrading HBx through the ubiquitin-proteasome pathway to inhibit HBV replication as a method of how innate immunity respond to HBx (142).

## 5 PRR agonists in HBV therapy

In light of the mechanisms underlying HBV’s evasion of innate immunity, targeting Pattern Recognition Receptor (PRR) pathways has emerged as a promising strategy for modulating antiviral responses in HBV infection. Several TLR agonists are currently being investigated in preclinical or early clinical stages for their potential therapeutic benefits.

GS-9620 (Vesatolimod), an orally administered TLR7 agonist, has been developed to restore antiviral immunity in CHB by activating host innate immune responses. Although GS-9620 does not directly target hepatocytes, it predominantly acts on peripheral blood mononuclear cells (PBMCs), particularly plasmacytoid dendritic cells (pDCs), leading to the induction of endogenous interferon type I (IFN-I) and interferon-stimulated genes (ISGs). This activation suppresses HBV RNA, DNA, and viral antigen expression through non-cytolytic mechanisms, without directly affecting cccDNA (149). *In vivo* studies show a dose-dependent effect, with increased innate immune activation creating a more favorable antiviral environment (150). Despite its promising safety profile and tolerability in CHB patients, clinical trials have not demonstrated significant reductions in HBsAg or serological conversion, suggesting that GS-9620 may be more effective as part of combination therapies rather than as a standalone curative treatment (151, 152). Further clinical trials, such as those investigating RO7020531, have highlighted the limitations of TLR7-mediated innate immune activation in humans. Although RO7020531 can induce interferon-related signaling *in vivo*, the activation plateaus within clinically tolerable doses, limiting its antiviral efficacy (19).

In contrast, JNJ-64794964 exemplifies the potential of TLR7 agonists to achieve a stronger antiviral effect. In murine models, robust activation of TLR7 leads to non-cytolytic suppression of HBV DNA and HBsAg to undetectable levels, with sustained viral control even after treatment cessation (153). However, these results depend on the intensity of immune activation and the experimental conditions, suggesting the need for careful consideration of their clinical relevance. Additionally, AIC649 represents a broader immunomodulatory strategy, targeting both TLR9-dependent and -independent pathways. Although monotherapy with AIC649 shows modest antiviral activity, its combination with nucleoside analogs enhances immune responses and extends viral suppression (154).

Together, these studies highlight the potential of PRR agonists to activate the innate immune system and suppress HBV replication. However, the magnitude and durability of these effects vary across agents and clinical settings, emphasizing the importance of evaluating PRR agonists primarily as components of combination therapy.

## 6 Conclusions and perspectives

The innate immune system provides the first line of defense against pathogenic infection, either through its direct antiviral effects or by indirectly initiating primary adaptive immune responses. In recent years, our understanding of the molecular mechanisms and pathways driving innate immune activation during HBV infection has advanced considerably. At the same time, we have also discovered more ways in which HBV evades the surveillance of the innate immune system. A thorough study of the relationship between the two can enable us to re-examine the current clinical treatment plans, identifying their shortcomings and prospects. In this review, we have thoroughly discussed the interaction relationships between innate immunity and the three components (DNA, RNA, and proteins) of HBV and have also listed some of the latest research findings in related fields.

In the DNA components of HBV, we focused on cccDNA and discussed in detail the survival strategies of HBV’s cccDNA and how it evaded the key DNA sensor, cGAS. Moreover, although studies have shown that sensors such as IFI16 and ABCF1 can recognize cccDNA, their antiviral effects are insufficient to completely eliminate the virus alone, because HBV actively limits their activity. Similarly, the deamination mediated by APOBEC3A requires sufficient interferon-induced signals to be effective. The immune escape environment created by cccDNA weakens and hinders the initial recognition of these receptors, leading to viral persistence. Currently, the mainstream treatment for viral DNA in clinical practice mainly relies on nucleoside analogs (NAs) to inhibit the synthesis and replication of viral DNA, targeting the viral reverse transcriptase and thereby inhibiting HBV DNA replication, but it rarely achieves a functional cure because cccDNA is difficult to be eliminated (155, 156). Recent advances highlight the potential of capsid assembly modulators (CAMs) as innovative antiviral strategies against HBV. GST-HG141, a novel CAM, functions by disrupting viral nucleocapsid formation and thereby impeding HBV DNA synthesis and pregenomic RNA (pgRNA) encapsidation. In a very recent (Aug 2025) Phase II randomized, placebo-controlled trial, GST-HG141 demonstrated significant antiviral efficacy in chronic hepatitis B patients with low-level viremia despite long-term nucleostide analog therapy. Treatment led to marked reductions in both HBV DNA and pgRNA, with favorable safety and tolerability profiles (157). These promising results suggest that GST-HG141 may represent a breakthrough therapeutic option and provide a new direction for targeting HBV at the DNA level.

Among the RNA components of HBV, we have listed some TLR receptors and elaborated on their possible innate immune roles in HBV infection through some basic experiments. Although there is still a lack of sufficient understanding of the interaction between TLR receptors and HBV, the conclusions drawn from some TLR receptor agonist trials are still valuable for clarifying the mechanism and for clinical drug development (158). We have focused on the interaction between the most crucial pgRNA in HBV RNA and the RIG-I receptor. RIG-I recognizes the 5’-triphosphate end of pgRNA and initiates downstream reactions. HBV, through means such as ADAR1 and TIAR, modifies pgRNA or directly reduces the activity of RIG-I to achieve the purpose of evading immunity. We have also introduced the new hotspot in HBV research: svRNA. With the advancement of technology, the research on svRNA may promote the understanding of the HBV pathogenesis and provide a new perspective for identifying potential biomarkers and therapeutic targets. In terms of the treatment methods targeting HBV RNA, the RNA-targeted therapies that have become a hot topic in recent years include anti-sense oligonucleotides (ASO) and RNA interference (RNAi), which can target the viral pregenomic RNA to reduce the expression and replication of viral proteins. These therapies use small interfering RNA (siRNA) to silence the HBV genes, thereby lowering the levels of DNA, RNA and proteins (159–162). At present, RNA-targeted therapies are still in the experimental stage. Their clinical efficacy is stronger as a monitoring tool than as a standalone therapy (163, 164). In-depth investigations of HBV RNA and its associated receptors will enhance the theoretical foundation of RNA-targeted therapeutic strategies and may facilitate future breakthroughs.

In the protein components of HBV, we have listed four main protein components in HBV infection and explained how they achieve their persistent infection by influencing the innate immunity. Targeting HBV key protein–host interaction interfaces and reinforcing host-intrinsic antiviral defenses hold significant promise for achieving a functional cure. Future research should focus on the optimization and clinical translation of HBx–DDB1 inhibitors and HBsAg–TAK1/TAB2 dissociation compounds, as well as explore the feasibility of enhancing host E3 ligases, such as TRIM25. Advancing these strategies may ultimately provide novel solutions to eradicate HBV infection and prevent virus-associated hepatocellular carcinoma.

Looking forward, although emerging therapies targeting HBV DNA, RNA, and proteins are promising, single-agent strategies remain insufficient to achieve a functional cure (148, 149). Combination therapies are likely to provide the breakthrough: integrating nucleostide analogs with novel agents such as capsid assembly modulators, RNA-targeted therapies, and immune modulators (e.g., HBx–DDB1 inhibitors or TLR agonists) could suppress viral replication, counter immune evasion, and restore host antiviral responses at multiple levels (165, 166). Future research should focus on optimizing multi-target regimens and identifying the most effective timing for combination interventions, ultimately paving the way toward a functional cure for HBV.
